# Opinion: Percutaneous electrical nerve field stimulation compared to standard medical therapy in adolescents with functional abdominal pain disorders

**DOI:** 10.3389/fpain.2024.1279946

**Published:** 2024-01-11

**Authors:** Adrian Miranda

**Affiliations:** Division of Pediatric Gastroenterology and Hepatology, Medical College of Wisconsin, Milwaukee, WI, United States

**Keywords:** PENFS, DGBIs, anti-cholinergic, functional abdominal pain, pediatric

In 1959, Dr. John Apley first published the book, The Child with Abdominal Pains, and described what he called “little bellyachers”. We now know these conditions as functional gastrointestinal disorders (FGIDs) or more recently, disorders of the gut-brain interaction (DGBIs). Despite the high prevalence of these disorders and the considerable negative impact on quality of life in children, no drug has ever received regulatory (FDA) approval for chronic abdominal pain in children or adolescents. Until now, no device had ever received clearance by the FDA, but several studies, including the one reported by Santucci et al. have consistently demonstrated the efficacy of PENFS for DGBIs in children (1–7). Pediatric gastroenterologists, like many other pediatric subspecialists, still resort to using “off-label” medications to try to alleviate pain, in hopes of improving the lives of children and their families.

In 1962 the FDA first approved amitriptyline for use as an anti-depressant in adults. This drug continues to be used off-label for other conditions, including childhood DGBIs. Interestingly, it is the anticholinergic class of medications that have consistently been used to treat DGBIs in both children and adults. The anticholinergic risk scale (ARS) ranks medications for anticholinergic potential on a 3-point scale (0, no or low risk; 3, high anticholinergic potential). Pediatric gastroenterologists have for decades, used level 3 anticholinergic drugs (ACDs) to treat DGBIs including functional abdominal pain, irritable bowel syndrome (IBS), cyclic vomiting syndrome and abdominal migraine, albeit with very little evidence for their efficacy, not to mention safety. Level 3 ACDs include amitriptyline, cyproheptadine, and “anti-spasmodics” like dicyclomine and hyoscyamine, to name a few (8). This paper by Santucci et al. calls into question several key issues regarding these commonly used drugs, primarily, do they really work, should we be considering other alternatives, and most importantly, are they safe for long-term use in children?

Acetylcholine is a prominent neurotransmitter in several brain regions and involved in learning and memory. Cholinergic neurons are essential in fine-tuning brain activity and maintaining the excitatory and inhibitory balance within neural circuits. ACDs competitively inhibit muscarinic receptors and inhibit their acetylcholine-mediated responses. Extensive evidence suggests that ACDs can negatively impact memory and cognition and increase the risk of dementia in adults, particularly amongst the elderly (9–17). A meta-analysis that included 26 studies found an association between anticholinergic burden and risk of cognitive decline and dementia in adults (12). In patients with schizophrenia, anticholinergic burden has been shown to be a determinant of cognitive ability and psychosocial treatment outcomes. In children, it is associated with an increased risk of delirium in the intensive care unit (18, 19). Few studies have been conducted in cognitively normal children (20, 21). Unfortunately, these studies are limited by very short follow-up periods ranging from a couple of weeks to 6 months. Theoretically, alterations to the cholinergic system could lead to neuronal dysfunction and impact the developing brain at a time of great neuroplasticity. In animals, amitriptyline was shown to have adverse effects on synaptic plasticity in the hippocampus (22). If there truly is an association between anticholinergic use and diminished cognitive functioning in aging adults, despite differences in the blood brain barrier, then the discussion needs to be extended to children and the developing brain. Because ACDs are highly discouraged in the elderly population, it is now common to see major insurance plans in the US caution against their use and provide alternatives for this population. In contrast, commercial plans often mandate providers to use step-therapy, requiring that these drugs be used as first-line in children and that they fail treatment prior to approving other more expensive therapies. The logic appears to be financially driven as these medications have been around for a half century with expired patents and therefore relatively inexpensive. Should we not be asking the question, how are these drugs affecting synaptic connections in brain regions that are dependent on acetylcholine? In other words, is it safe to block neuronal synapses in the developing brain by blocking the effects of an important neurotransmitter and potentially altering neuroplasticity. The effect of these medications on the gut-brain axis is also unknown.

Acetylcholine is the primary neurotransmitter of the vagus nerve, which represents the main conduit for the parasympathetic nervous system and oversees many important bodily functions including immune response, digestion, heart rate and even control of mood. Most of us have encountered patients who develop worsening symptoms after starting medications only to adjust the dose or discontinue them. One must question whether this could be related to worsening vagal insufficiency which has been demonstrated in children with DGBIs (23, 24). In addition, blocking muscarinic-dependent vagal activation with ACDs could lead to worsening inflammation. Already, an imbalance of proinflammatory and anti-inflammatory cytokines has been found in adults with IBS (25, 26). The cholinergic anti-inflammatory pathway (CAP) is a classic neuroimmune pathway that facilitates crosstalk between the autonomic nervous system (ANS) and the immune system primarily through the vagus nerve and its pivotal neurotransmitter, acetylcholine. This pathway plays an important role in the anti-inflammatory response through activation and regulation of immune cells. Sanghavi et al., recently described, in a large-scale prospective study, the strong association between anticholinergic burden and inflammation. In that study, the anticholinergic burden scores were independently associated with significant increases in fibrinogen, CRP, TNF-α and IL-6 (27). It is then plausible that several separate mechanisms related to ACDs could be responsible for potentially adverse long-term outcomes. Sixty years later, we still do not have an adequate understanding of who not to treat with these medications and what the ideal duration of treatment should be? Our literature makes little or no mention of the potential adverse effects of these medications on key systems, including the developing brain. This is critical since long-term treatments in children are likely to have a long-lasting impact. Pharmaceutical companies have, for the most part, ignored this population of pediatric patients and society guidelines for treatment of DGBIs in children had not been updated in over 17 years.

Compelling data regarding the efficacy of these drugs would improve the balance between these benefits and risks. Unfortunately, the data regarding their effectiveness for children with DGBI is weak, at best. Not only are more studies needed to demonstrate efficacy, but more are needed to show safety in children. The most common side effects that are shared with families prior to prescribing include sedation, weight gain, suicidal ideation, and cardiac rhythm disturbances, but no studies have investigated the long-term risks that have been found in adults. While the medications could potentially have harmful side effects, without short and long-term studies we simply don't know. Without the proper studies, there is no way of knowing if the increased risk of dementia or Alzheimer's disease with ACDs in adults has any parallels in children. It is very reminiscent of our subspecialty believing that the majority of children with encopresis outgrow the condition as adults, only to find later that a large majority have persistent symptoms long into adulthood.

Few studies have compared treatments head-to-head and while the current study has limitations, Santucci et.al. should be commended for taking on this task. Until we know more about their impact on the developing brain, the ANS and immune system, we have a responsibility to prescribe these medications in children judiciously, particularly when there is evidence of harmful or detrimental effects in adults. Clearly, there is a role for pharmacotherapy, but duration of treatment should be discussed as should potential alternatives. These include therapies with proven benefit and few side effects that enhance the body's ability to self-regulate and restore homeostasis such as PENFS or psychological therapies including gut-directed hypnotherapy, CBT, or biofeedback. Consideration should also be given to peppermint oil, safer OTCs or natural supplements and dietary changes. We also cannot neglect the benefits of lifestyle modification such as exercise and improved sleep hygiene. Future interventions may also involve artificial intelligence (AI) that could, for example, make general pediatricians and families feel more comfortable with the diagnosis of DGBIs without having to refer to the sub-specialist for additional testing. Also, the use of virtual reality as a treatment modality could potentially help “reset” brain pathways that are involved in pain processing. As physicians, we have a responsibility to offer the best and safest therapies to the patients and families we care for. The time has come to question and study whether prescription, off-label medications are safe in children with DGBIs and how best to use them. Until now, polypharmacy with a high anticholinergic burden was believed to be a problem isolated to the elderly population. However, it is not uncommon to see children present to our clinics on multiple medications that include antidepressants, alpha 2 delta ligands, mood stabilizers, anti-psychotics, and stimulants. While some of these have been major therapeutic breakthroughs for our patients, our responsibility as pediatricians is to try and minimize polypharmacy and above all, limit the potential impact on the developing brain since it is likely we will not know the long-term impact for years to come.
